# A novel network with enhanced edge information for left atrium segmentation from LGE-MRI

**DOI:** 10.3389/fphys.2024.1478347

**Published:** 2024-12-10

**Authors:** Ze Zhang, Zhen Wang, Xiqian Wang, Kuanquan Wang, Yongfeng Yuan, Qince Li

**Affiliations:** ^1^ School of Computer Science and Technology, Harbin Institute of Technology, Harbin, China; ^2^ Department of Spinal Surgery, Zibo Central Hospital, Zibo, China; ^3^ Department of Cardiology, Zibo Central Hospital, Zibo, China

**Keywords:** left atrium segmentation, edge information enhancement, deep convolutional network, weighted loss function, LGE-MRI

## Abstract

**Introduction:**

Automatic segmentation of the left atrium (LA) constitutes a crucial pre-processing step in evaluating heart structure and function during clinical interventions, such as image-guided radiofrequency ablation of atrial fibrillation. Despite prior research on LA segmentation, the low contrast in medical images exacerbates the challenge of distinguishing various tissues, rendering accurate boundary delineation of the target area formidable. Moreover, class imbalance due to the small target size further complicates segmentation.

**Methods:**

This study aims to devise an architecture that augments edge information for LA segmentation from late gadolinium enhancement magnetic resonance imaging. To intensify edge information within image features, this study introduces an Edge Information Enhancement Module (EIEM) to the foundational network. The design of EIEM is grounded in exploring edge details within target region features learned from images. Additionally, it incorporates a Spatially Weighted Cross-Entropy loss function tailored for EIEM, introducing constraints on different regions based on the importance of pixels to edge segmentation, while also mitigating class imbalance through weighted treatment of positive and negative samples.

**Results:**

The proposed method is validated on the 2018 Atrial Segmentation Challenge dataset. Compared with other state-of-the-art algorithms, the proposed algorithm demonstrated a significant improvement with an average symmetric surface distance of 0.684 mm and achieved a commendable Dice coefficient of 0.924, implicating the effectiveness of enhancing edge information.

**Discussion:**

The method offers a practical framework for precise LA localization and segmentation, particularly strengthening the algorithm’s effectiveness in improving segmentation outcomes for irregular protrusions and discrete multiple targets. Additionally, the generalizability of our model was evaluated on the heart dataset from the Medical Segmentation Decathlon (MSD) challenge, confirming its robustness across different clinical scenarios involving LA segmentation.

## 1 Introduction

Atrial fibrillation stands as the most prevalent sustained cardiac arrhythmia, carrying an elevated risk of heart failure, vascular embolism, and sudden death. Timely diagnosis and intervention are pivotal in enhancing patients’ cardiac function and reducing mortality rates. Late gadolinium enhancement magnetic resonance imaging (LGE-MRI) provides a comprehensive view of cardiac structural morphology and function. The assessment of the LA in LGE-MRI images holds crucial clinical significance, including planning and guidance of atrial fibrillation ablation, postoperative follow-up studies, fibrosis quantification, and biophysical modeling (Aschauer et al., 2016; Khurram et al., 2016). However, the small left atrial cavity, enclosed by the thin atrial wall and featuring complex anatomical structures such as irregular protrusions and discrete multiple targets (Maceira et al., 2010; Wang et al., 2019; Xiong et al., 2021), poses challenges. Moreover, adjacent anatomical structures, such as the left ventricle, often exhibit similar image intensities, making accurate identification more difficult. While manual segmentation could reconstruct and explore the atrial structure, it typically necessitates specialized domain knowledge and incurs high labor costs (Oakes et al., 2009). Additionally, manual atrium segmentation is time-consuming, error-prone, and highly subjective (Petitjean and Dacher, 2011; Caudron et al., 2012). Therefore, the development of an automatic segmentation method with high precision for the LA holds substantial scientific significance and practical value as an auxiliary tool for medical diagnosis, treatment planning, prognosis, and related applications. Traditional segmentation methods, based on regional growing (Karim et al., 2008) and atlas guiding (Zuluaga et al., 2013), encounter limitations due to poor image quality and individualized variations in anatomical structures. In contrast, deep learning methods, renowned for their exceptional segmentation accuracy, possess the ability to automatically learn image features and have extensive applications in image segmentation (Litjens et al., 2017). The U-Net network (Ronneberger et al., 2015), a variant of Fully Convolutional Network (FCN) (Long et al., 2015), has been widely adopted for medical image segmentation since 2015. Building upon the success of 2D U-Net, 3D deep learning networks, such as 3D U-Net (Çiçek et al., 2016) and V-Net (Milletari et al., 2016), have been developed to directly process 3D volumetric data and generate 3D volumetric segmentation results. In the domain of atrial segmentation, deep learning has been the focal point of comprehensive research (Drozdzal et al., 2016). modified the network structure by introducing short-skip connections to FCN, achieving notable segmentation results without post-processing operations (Yang et al., 2020). proposed a joint segmentation method, combining spatial consistency from multiple views with a recursive attention module for LA and scar segmentation in 3D LGE-MRI images (Wong et al., 2022). Proposed a novel GCW-UNet architecture, incorporating Gaussian blur and channel weight neural network for automatically segmenting the left atrial region in MRI images of patients with left atrial enlargement (Uslu and Bharath, 2023). Presented a quality control method based on the multi-view network TMS-Net. The approach significantly improves noise robustness and run-time quality estimation for cardiac MRI segmentation through an innovative design featuring a single encoder and three decoders (Raj Singh et al., 2023). Introduced ARW-Net, a deep learning-based segmentation approach with attention-guided residual links and upgraded deep supervision, showcasing its potential as an outstanding solution for automated and generalized cardiac segmentation. Numerous studies have integrated deep learning methods with traditional approaches or incorporated prior knowledge into deep learning networks to obtain more anatomically reasonable segmentation results. For instance, (El Jurdi et al., 2020), integrated position and shape information into the convolutional layers of the model, guiding the model to identify the target structure’s location and fine-tuning network parameters under a fully supervised learning framework.

Despite the significant progress achieved by deep learning methods in atrial image segmentation, challenges persist, particularly in data imbalance and effectively segmenting blurred edges. In cardiac MRI images, the LA or its margins serve as small structures, contributing to class imbalance issues within the data due to their limited size. Approaches to addressing class imbalance encompass techniques such as image cropping and cascaded networks. For instance (Xiong et al., 2020), employed two continuous Convolutional Neural Networks (CNNs) for atrium segmentation. The first CNN identifies the region of interest, while the second CNN is utilized for target structure segmentation. Based on the 3D U-Net (Vesal et al., 2020), developed a two-stage architecture encompassing coarse and fine segmentation, achieving end-to-end learning. In contrast to cropping or cascaded network methods (Kausar et al., 2021), addressed class imbalance by leveraging prior knowledge and posterior handling operations, utilizing a dense V-Net for segmentation, and fine-tuning parameters. In addition (Kausar et al., 2023), proposed a 3D shallow residual segmentation network based on the 3D multi-scale residual learning structure, introducing a composite loss function and parameter adjustment to tackle class imbalance in medical image datasets without pre-processing and post-processing. Despite successful LA segmentation, these methods exhibit limitations, such as information loss in cropping or cascaded network approaches, redundancy in two-stage architectures, challenges in ensuring model robustness, susceptibility to subjective judgments and empirical influences in designing composite loss functions and adjusting parameters. Additionally, efforts have been made to tackle the challenge posed by the indistinct edges of target structures (Huang et al., 2022). applied the distance map associated with the target structure’s edge as a weight map and utilized a two-stage network to improve LA segmentation performance (Uslu et al., 2022). designed a multi-task segmentation network that integrated edge information of the image into decoding modules of multiple scales. Nevertheless, these methodologies did not fully leverage the available edge information, limiting their effectiveness in addressing the challenges posed by blurred boundaries.

Therefore, to improve the performance in atrial boundaries, this research introduces an EIEM capable of optimizing segmentation by learning and reinforcing boundary information of the target structure, accompanied by a specifically designed Spatially Weighted Cross-Entropy (SWCE) loss function that constrains the module. The contributions of this work can be summarized as follows:

• A novel EIEM was developed to enhance the edge information for image segmentation by integrating edge features with region features. Channel attention modules and multi-scale structural feature fusion are incorporated for edge information learning, enhancing the model’s attention to crucial edge feature channels and the model’s capacity to capture diverse-scale structures and details.
• In this study, we innovatively designed a SWCE loss function tailored to the EIEM to address the class imbalance in the dataset. This loss function incorporates dynamic weighting for positive and negative samples, allowing for adaptive adjustments. Combined with image cropping, the customized loss function effectively mitigates class imbalance in a flexible and data-driven manner.
• The SWCE loss function adapts to the significance of each area through its distance-sensitive weighting scheme, aiming to effectively leverage edge details and alleviate challenges posed by fuzzy edges in segmentation. Supplemented by the Cross-Entropy loss linked to the target region segmentation network, the SWCE loss encourages the model to prioritize edge regions and enhances flexibility.
• The proposed framework is validated on the 2018 Atrial Segmentation Challenge dataset, demonstrating superior performance compared to state-of-the-art methods, especially regarding average symmetric surface distance. The integration of edge constraints improves segmentation outcomes, particularly for irregular protrusions and discrete multiple targets.

The paper is organized as follows: Section 2 provides a detailed introduction to our model; Section 3 outlines the experimental methodology; Section 4 discusses the experimental results; and Section 5 concludes the paper.

## 2 Materials and methods

### 2.1 Datasets

The datasets utilized in the proposed method are sourced from two distinct collections:

2018 Atrial Segmentation Challenge dataset (Xiong et al., 2021): The dataset used comprises 100 3D LGE-MRI images. While the dataset included additional test cases, only the 100 training cases were utilized in our study as they were the primary dataset provided for training and validation during the challenge. These cases were considered sufficient to meet the experimental requirements. Each set of images consists of an original image and a corresponding ground truth (GT) label. The original images cover the full LA, and the GT labels include the intact left atrial cavities and part of the pulmonary veins annotated by domain experts. The spatial resolution of these images is 0.625 × 0.625 × 0.625 mm³. Each 3D volume comprises 88 slices along the *Z*-axis, and the image dimensions are either 640 × 640 or 576 × 576 pixels. The grayscale values in the original images range from 0 to 255. For the GT labels, the grayscale value is 0 or 255, and a grayscale value of 0 denotes the background class, while a value of 255 designates the area constituting the GT label.

MSD heart dataset (Simpson et al., 2019; Antonelli et al., 2022): This dataset consists of 20 annotated MRI scans, each containing approximately 100 2D image slices, capturing the entire heart at a single cardiac phase. The images were acquired under free-breathing conditions, with ECG gating, using a 1.5T Achieva scanner. The voxel resolution for these scans is 1.25 × 1.25 × 2.7 mm³. Labeling of the left atrium, including the left atrial appendage, mitral plane, and portal vein end points, was performed using an automated tool, followed by the expert’s manual corrections to ensure high accuracy. A key characteristic of this dataset is the small sample size combined with significant variability across the images, which was used to evaluate the generalization performance of the model trained on the 2018 Atrial Segmentation Challenge dataset.

### 2.2 Pre-processing

Since the MRI images show that the proportion of the LA is small, there is a class imbalance between the foreground class, composed of the LA, and the background class, consisting of other anatomical structures. The learning process tends to focus on the large background class, resulting in poor segmentation of the LA. Moreover, some images consist of inconsistent dimensions. Therefore, based on the positional information of the LA, in this study, the images are first center-cropped to 300 × 300 pixels (from the 2018 Atrial Segmentation Challenge dataset) or 130 × 130 pixels (from the MSD heart dataset) to increase the proportion of the region of interest in the image and then resized to fit the same size of 256 × 256, aligning with the network’s input requirements. The difference in cropping sizes arises from variations in the proportions of the LA within the images, with the cropping sizes being roughly selected to ensure that the background is reduced while retaining the entire LA in all cases. Moreover, data augmentation is applied to the training set through a randomized combination of transformations, including rotation, translation, and scaling, in line with the conditions that may be encountered during medical image acquisition. This operation introduces diversity into the data, augmenting the presence of noisy data, while also mitigating the impact of variations in cropping sizes. The inclusion of a variety of data and the introduction of additional noisy data can enhance the model’s robustness and generalization capabilities.

For both datasets, the data is split into 60% for training, 20% for validation, and 20% for testing. Each data set used for network training consists of images, corresponding GT labels, and generated edge images. Both GT labels and generated edge images are binary, with foreground regions set to 1 and background regions set to 0. The erosion operation is utilized to extract the edges from the GT labels.

### 2.3 Edge information enhancement segmentation network

In this study, the 2D U-Net network serves as the backbone architecture for learning the region features of the target anatomy. The U-Net network comprises nine blocks, each with two continuous 3 × 3 convolution layers. Following pre-processing, the input images are fed into the network. After two successive 3 × 3 convolutions in the encoder block, down-sampling is performed through the max-pooling process since the max-pooling operation can retain more texture information compared to the average-pooling. The decoder block also contains two successive 3 × 3 convolution layers, followed by up-sampling achieved through deconvolution. The batch normalization and dropout layers are used in the network as regularization techniques to mitigate overfitting. The Relu activation function is applied in feature extraction layers, while the sigmoid activation function is employed in classification layers.

The EIEM concentrates on learning edge features of the target anatomy from the backbone network, while also integrating the learned edge details back into the backbone network. Initiating from input images, the network’s learning process diverges into two distinct directions: learning region features through the backbone network and extracting edge features through the EIEM, which complement each other. The network produces two main outputs: the predicted label images and the predicted edge images for the LA. The final segmentation results are obtained by post-processing the predicted label images by extracting the maximum connected component.

#### 2.3.1 Edge Information Enhancement Module, EIEM

**FIGURE 1:**
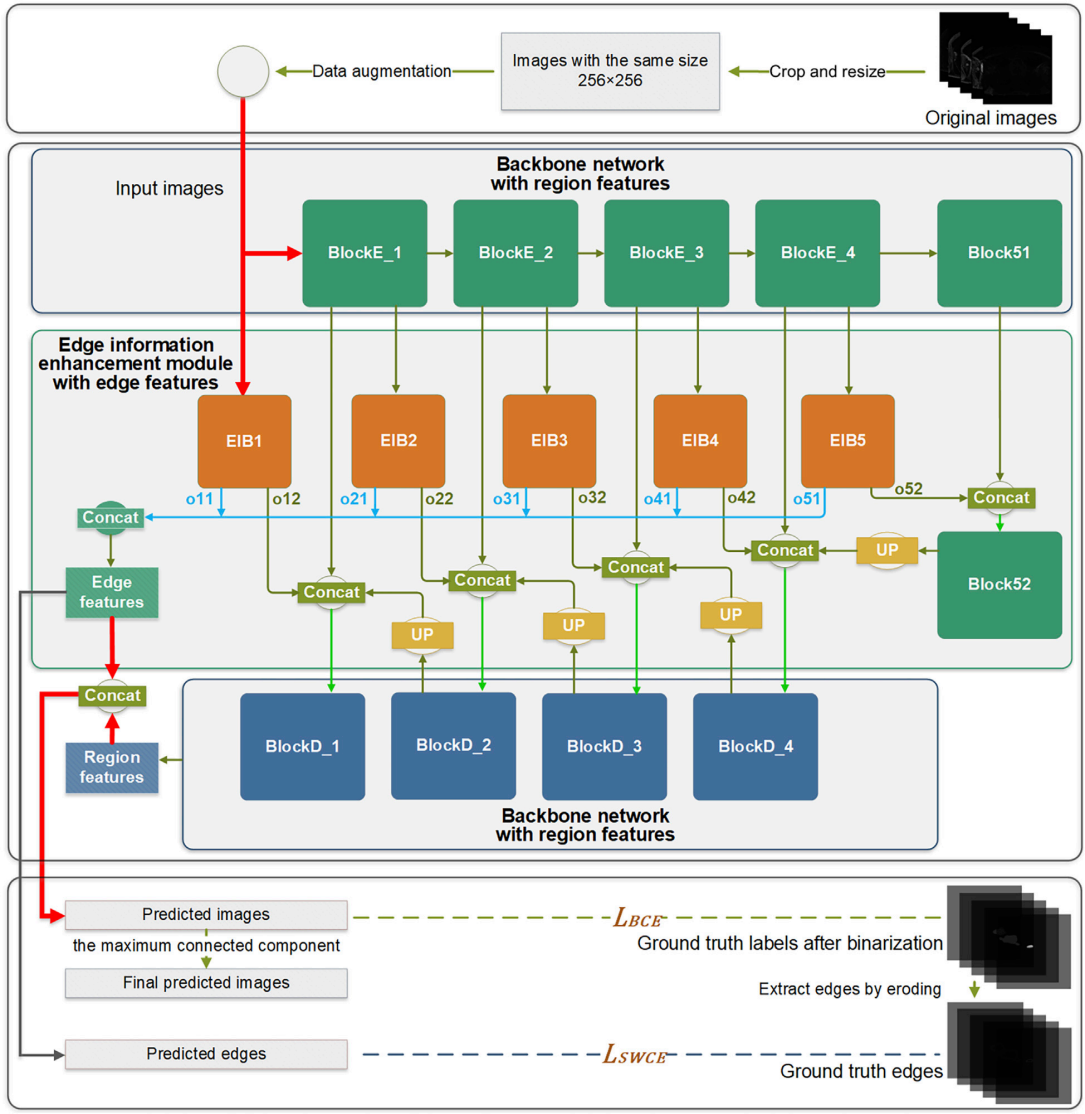
The overall architecture of the proposed method.

Edge information plays a critical role in accurately delineating the boundaries of the target and improving overall segmentation accuracy. To learn and enhance the edge information of the target structure, an EIEM is introduced, as illustrated in Figure 1. The EIEM first learns edge features from the backbone network at multiple scales and integrates these multi-scale features. These learned edge features are then fed back into the backbone network for additional reference. The EIEM takes the input image and region feature maps as inputs, and the outputs are edge feature maps of the target structure. The input image, as well as outputs of the first four blocks in the backbone network, serve as inputs for the branches of EIEM, constituting a total of five side branches. The first side branch (EIB1) directly takes the original image as input and perceives global image information, which can capture finer-grained features. These low-level features typically contain more details and texture information, which helps to improve the model’s sensitivity to input images and enhance edge feature extraction. This branch integrates with the backbone network by receiving input directly from the image and providing comprehensive edge details that enhance the global context for subsequent processing stages. The inputs of other side branches (EIB2-5) receive region features of the target anatomy at different scales from the backbone network. Two sequential 3 × 3 convolution operations are subsequently employed to extract the edge features from the multi-scale region features. The resultant feature maps are then up-sampled to match the input image size, aligning with region features from the output of the backbone network. Within this submodule, regularization constraints, including batch normalization and dropout layers, are applied.

Within the edge information block (EIB), channel attention (Hu et al., 2018) is applied to enhance the network’s learning capacity for edge details by focusing on channels containing crucial edge-related information of the target structure. The dynamic adjustment of channel weights by channel attention enables a selective suppression of channels deemed less relevant to edge segmentation, thereby reducing the computational burden associated with processing redundant information. This mechanism is integrated into the backbone network by optimizing feature representations before combining them with the region features from the backbone. Due to its superior performance and low computational cost, channel attention significantly enhances the network’s ability to capture critical edge information while reducing computational redundancy.

**FIGURE 2:**
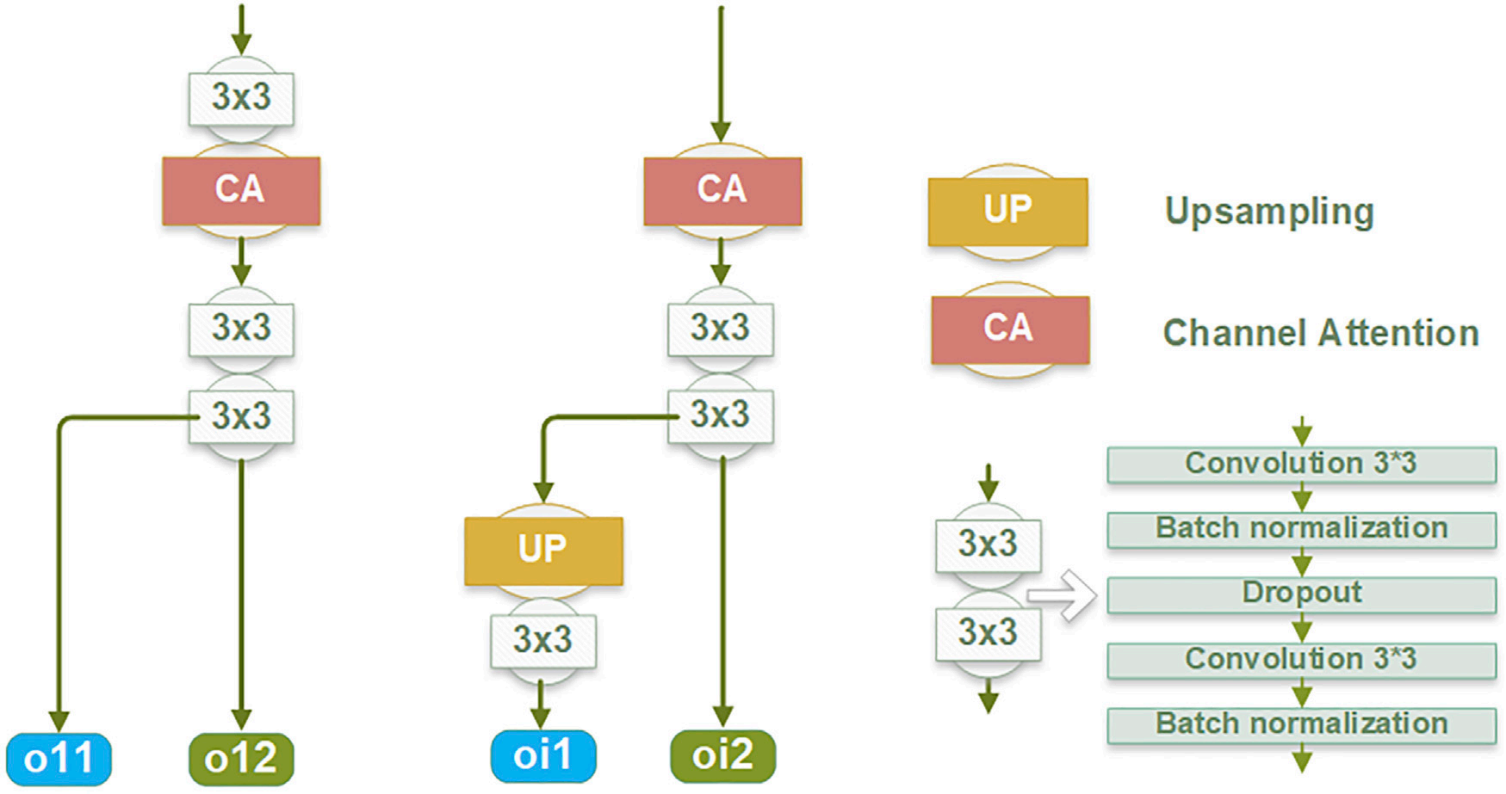
The structure of the edge information block (EIB).

Each EIB yields two outputs: the edge features at the original image size (oi1) and the edge features before undergoing up-sampling (oi2), as depicted in Figure 2. The oi1 outputs are fused and processed to obtain the final predicted edge images, constrained by the SWCE loss to optimize the EIEM’s performance in edge segmentation. Concurrently, the fused edge features are combined with the region features learned by the backbone network to enhance the network’s overall segmentation performance on the target, constrained by Binary Cross-Entropy (BCE) loss. Furthermore, the oi2 outputs are integrated with the region features from the decoder parts of the backbone network, providing additional edge information for learning atrial region features and ensuring the preservation of sensitivity to the edge throughout the decoding process. These comprehensive integration and fusion processes with the backbone network, guided by SWCE and BCE losses, ensure the model’s effectiveness in capturing and delineating edges and reinforce the overall accuracy of the model’s segmentation outputs.

#### 2.3.2 Loss functions

The network incorporates two loss functions: the Binary Cross-Entropy Loss ( $L_{BCE}$ ) associated with the target region segmentation network and the Spatially Weighted Cross-Entropy Loss ( $L_{SWCE}$ ) pertaining to the EIEM. The comprehensive loss (*L*) of the network is the weighted summation of these two losses, expressed by the formula (Equation 1):

$$L=w_{a}L_{BCE}+w_{b}L_{SWCE}$$ (1)

where $w_{a}$ and $w_{b}$ are set the values 0.5 each, representing the weighted coefficients for the two losses.

BCE loss is selected for its ability to achieve a better balance between sensitivity and specificity in segmentation results, exhibiting greater stability and a propensity to converge more readily toward the global minimum throughout the optimization process. The BCE loss function of the network is as follows (Equation 2):

$$L_{BCE}=-y*\log\left(\hat{y}\right)-\left(1-y\right)*\log\left(1-\hat{y}\right)$$ (2)

where y denotes the value of GT labels and $\hat{y}$ denotes the value of predicted images.

The left atrial edges occupy only a tiny region in a thoracic LGE-MRI image, with the background class covering a significant proportion of the data. Therefore, the SWCE loss function of the network is designed based on the weighted binary cross-entropy loss function to ensure a balanced impact of foreground and background classes on network learning. Further, the SWCE loss function introduces additional parameters to dynamically modulate the importance of pixels based on their spatial characteristics and mitigate class imbalance through dynamic weights assigned to positive and negative samples during computation. The formulation of the loss function is defined as follows (Equation 3):

$$L_{SWCE}\left(y,\hat{y}\right)=-\frac{1}{N}\sum_{i=1}^{N}\left[c\cdot y_{i}\cdot \log\left({\hat{y}}_{i}\right)+\left(1-y_{i}\right)\cdot \log\left(1-{\hat{y}}_{i}\right)\right]\cdot w_{i}$$ (3)

where N represents the total number of pixels in the image, $y_{i}$ denotes the GT label for pixel i , ${\hat{y}}_{i}$ represents the predicted probability for pixel i , and $w_{i}$ signifies the spatial weight of the pixel i . The spatial weight $w_{i}$ is computed based on the pixel’s proximity to the edge. $w_{i}$ and $y_{i}$ are given by the following expressions ( Equations 4, 5):

$$w_{i}=\left\{\begin{array}{ll} \;a, & if\;y_{i}=1\\ \;b+\frac{1}{d\_to\_e\left(i\right)}, & if\;y_{i}=0\text{ and }\exists j,y_{j}=1\\ \;b, & if\;\forall i,{\;y}_{i}=0 \end{array}\right.$$ (4)

$$y_{i}=\left\{\begin{array}{c} \;1,if\;d\_to\_e\left(i\right)\leq d-1\\ \;0,otherwise \end{array}\right.$$ (5)

where $d\_to\_e\left(i\right)$ is the Euclidean distance from pixel i to the nearest edge pixel. In Equation 5, the method for thickening the edge inward involves an erosion operation, with the default that pixel i is inside the edge. The parameters *a*, *b*, *c*, and *d* in Equations 3–5 play crucial roles in the SWCE loss function, governing edge weighting, weight decay rate, class-wise weighting, and edge range, with each serving specific purposes in improving segmentation performance. These parameters are designed to address challenges related to boundary precision and handling class imbalance.

Firstly, parameter *a* serves as the edge weighting and is introduced to enhance the model’s sensitivity to edges. Assigning higher weights to edge pixels encourages the model to focus more on capturing fine boundary details. This is particularly important in cases where the target structure is small, complex, or irregularly shaped, as these challenging characteristics make it more difficult for the model to capture and differentiate fine boundary details. In such scenarios, the model must be even more sensitive to subtle edge variations, making the role of parameter *a* crucial for improving segmentation performance.

Secondly, parameter *b* governs the weight decay rate based on the pixel’s distance from the edge. This helps manage the class imbalance between the large background and the small foreground regions by reducing the influence of background pixels farther from the edge. Combining the reciprocal of the $d\_to\_e\left(i\right)$ with parameter *b* achieves a weight decay mechanism, assigning higher weights to pixels closer to the edges and lower weights to those farther away during loss computation, thereby emphasizing pixels near the boundary. Since pixels near the boundary often contain critical transitional information between different regions, focusing on them allows the model to capture fine boundary details more accurately. When *b* is set to a smaller value, the sensitivity of loss weights to distance increases, prompting the model to focus more on pixels near the edges and emphasize the learning of edge details. Conversely, with a larger value of *b*, the sensitivity of loss weights to distance decreases, allowing the model to process the entire image smoothly. This design enables flexible adjustments to the model’s focus on different regions of the image, balancing the importance of pixels in the image and enhancing its ability to learn edge information.

Thirdly, parameter *c* functions as the class-wise weighting to address the class imbalance, helping to balance the contributions of positive and negative samples during training. By tuning *c* appropriately, the SWCE loss function adjusts the relative importance of positive and negative samples, ensuring the model effectively learns from both classes. This adjustment leads to a more balanced performance across classes.

Lastly, parameter *d* denotes the edge range. By expanding *d* - 1 layers inward, the annotated edge region is broadened, providing a more extensive context of the edge features for supervision, which aids in learning more robust edge representations. A larger *d* value extends the edge region, allowing the model to capture more contextual information around the boundary, which is beneficial for learning the structural characteristics of the edge. However, an excessively large *d* value may introduce unnecessary complexity. Therefore, the value of *d* should strike a balance between capturing sufficient context and avoiding overcomplication. Additionally, *d* will further affect the calculation of w in Equation 4 due to its impact on the y-value of pixels.

Together, these parameters enable the SWCE loss function to focus on edge regions, manage class imbalance, and provide flexibility in how different areas of the image are weighted during training. Their careful selection and tuning, as demonstrated in our ablation studies, contribute to optimizing the model’s performance in image segmentation tasks, enhancing its fine-grained perception of target structures and segmentation accuracy.

#### 2.3.3 Metrics

The proposed method uses the Dice coefficient (DC), Jaccard coefficient (JC), and average symmetric surface distance (ASSD) as metrics to assess the validity of the results.

The DC quantifies the overlap between GT labels and predicted images, with values between 0 and 1. A higher DC signifies a larger overlapping area, indicating a better outcome. The formula for the DC is as follows (Equation 6):

$$D\left(A,B\right)=\frac{2\cdot \left|A\cap B\right|}{\left|A\right|+\left|B\right|}$$ (6)

where A represents the GT labels, B denotes the predicted images, $\left|A\cap B\right|$ signifies the intersection of A and B . $\left|A\right|$ and $\left|B\right|$ denote the sum of the voxel values of the images. The JC is a metric used to assess the similarities and differences between GT labels and predicted images. A higher JC value indicates a greater similarity between the two. The formula for the JC is as follows (Equation 7):

$$J\left(A,B\right)=\frac{\left|A\cap B\right|}{\left|A\cup B\right|}$$ (7)

where $\left|A\cap B\right|$ represents the intersection of A and B , and $\left|A\cup B\right|$ denotes the union of A and B . The ASSD is chosen as the metric for assessing the segmentation results in terms of distance. The unit of ASSD is in millimeters, quantifying the average distance between two surfaces in the images. A smaller ASSD value indicates a closer alignment between GT labels and predicted images, reflecting a better segmentation result. The formula for the ASSD is as follows (Equation 8):

$$ASSD\left(A,B\right)=\frac{ASD\left(A,B\right)+ASD\left(B,A\right)}{2}$$ (8)

in which Equation 9 is given by the following expression:

$$ASD\left(A,B\right)=\frac{\underset{a\in A}{\Sigma }min_{b\in B}d\left(a,b\right)}{\left|A\right|}$$ (9)

where $ASD\left(A,B\right)$ denotes the Average Surface Distance between volumes A and B , and $d\left(a,b\right)$ represents the Euclidean distance between pixels a and b . $\left|A\right|$ signifies the number of surface voxels in volume A .

To further evaluate the performance of the proposed method, we compute the mean and standard deviation (std) of the metrics (DC, JC, ASSD) across test cases. The mean reflects the average performance of the segmentation, providing a central tendency across cases. Meanwhile, the standard deviation quantifies the consistency or variability of the segmentation results. A smaller standard deviation suggests more stable performance, while a larger one indicates greater variability across test cases. Together, the mean and standard deviation offer insights into not only the overall effectiveness of the segmentation method but also its stability across various conditions.

## 3 Experiments and results

Due to the challenges posed by edge blurring and the similarity in grayscale values of adjacent tissues in medical images, the segmentation of boundary details of the target structure presents a challenging task. In response to this challenge, the study introduces an edge information enhancement design and conducts multiple sets of ablation experiments to demonstrate the effectiveness of the proposed method.

Our model was implemented using the Keras framework (version 2.11.0) and operates on a workstation equipped with an NVIDIA GeForce RTX 3090 GPU. The operating system is Ubuntu 22.04, and the Python environment uses version 3.8. Additionally, CUDA 11.2 and cuDNN 8.1 are employed for GPU acceleration. We employed the Adamax optimizer with polynomial decay to adjust the learning rate during training, starting with an initial value of 0.001. The model was trained for 200 epochs, with the DC on the validation set monitored throughout the training process. The DC on the validation set continuously improved and stabilized around the 200th epoch. As the final model, we selected the parameters that achieved the highest DC on the validation set, ensuring optimal segmentation performance during testing. For further details on the code and environment setup, please refer to the following GitHub repository: https://github.com/PencilSC/EIEM, where we provide the code and necessary configurations to facilitate reproducibility.

### 3.1 Performance evaluation of the proposed framework

To assess the performance of the proposed model, this study compares it with several state-of-the-art segmentation models on the 2018 Atrial Segmentation Challenge dataset, including nnU-Net (Isensee et al., 2021). The quantitative comparison results, based on three evaluation metrics, are presented in Table 1. In contrast to the other methodologies enumerated in Table 1, the presented approach demonstrates superior performance regarding the ASSD. Meanwhile, the Dice and Jaccard coefficients of this study achieve results comparable to other state-of-the-art methods.

**TABLE 1:** Performance comparison between the proposed method and other methods for LA segmentation based on DC, JC, and ASSD with ± std following the values.

Method	DC (%)	JC (%)	ASSD (mm)
2D U-Net	91.36 ± 2.14	84.16 ± 3.56	0.832 ± 0.253
Chen et al. (2019)	90.1 ± 3.0	82.2 ± 6.0	1.04 ± 0.32
Yang et al. (2019)	92.24	85.64	1.490
Bian et al. (2019)	92.83	**86.69**	1.496
Isensee et al. (2021)	92.80 ± 1.74	86.62 ± 3.02	0.942 ± 0.741
Uslu et al. (2022)	92.1 ± 1.8	85.5 ± 1.3	0.862 ± 0.237
Huang et al. (2022)	**94.1**	−	0.82
Ocal. (2024)	91.49	−	−
The proposed method	92.43 ± 1.36	85.96 ± 2.35	**0.693** ± **0.152**

Bold values indicate the highest performance within each column.

Although the algorithm proposed in this study ranks fourth in DC and third in JC, the experimental results show that our algorithm yields slightly lower performance than the methods proposed by Huang et al. (2022), Bian et al. (2019) in terms of Dice and Jaccard coefficients, with only a small difference in values. Meanwhile, our proposed algorithm achieved a significantly improved ASSD result of 0.693 compared to Huang et al.'s 0.82 (the best result of assessed algorithms) and Bian et al.'s 1.496. This enhancement indicates that our algorithm can more accurately capture the manually segmented LA. Compared to other methods, our proposed algorithm enhances boundary information, resulting in more accurate segmentation outcomes, which is crucial for clinical diagnosis and treatment planning. Accurate boundary segmentation can assist clinicians in identifying pathological areas better and provide support for surgical navigation and treatment efficacy evaluation. The lack of notable differences in Dice and Jaccard coefficients may be attributed to factors such as dataset characteristics, data pre-processing, algorithm architecture, and implementation details.

### 3.2 Ablation and analysis

#### 3.2.1 Number of branches

The encoder progressively reduces the size of the feature maps through multiple pooling operations. In the shallow layers of the U-Net, where the resolution of feature maps is higher, fine details such as edge information are preserved. To investigate the impact of varying the number of side branches on the experimental outcomes, ablation experiments were conducted, specifically targeting the number of lateral branches to assess the method’s performance. Starting with the shallow layers, the encoder part utilizes one to five branches for learning edge information. These results illustrate the impact of varying lateral branch quantities on method performance, assessed via DC, JC, and ASSD, as presented in Table 2. Increasing the number of branches from one to five shows improved segmentation performance, especially in capturing fine edge details, as evidenced by higher DC and JC values and lower ASSD values. The incorporation of channel attention modules and multi-scale structural feature fusion enhances the model’s ability to focus on crucial edge feature channels and capture diverse-scale structures and details. Therefore, despite the progressive reduction in resolution as the number of branches increases, fine details such as edge information are preserved. Deeper layers capture high-level semantic information, while shallow layers retain fine-grained details. By incorporating edge information at multiple scales, the proposed method effectively utilizes both high-level semantic context and low-level fine details, leading to more accurate segmentation results.

**TABLE 2:** Ablation experiments assessing the impact of varying lateral branch quantities on the proposed method’s performance, evaluated through DC, JC, and ASSD with ± std.

Method	DC (%)	JC (%)	ASSD (mm)
EIEM (5)	**92.43** ± **1.36**	**85.96** ± **2.35**	**0.693** ± **0.152**
EIEM (4)	92.14 ± 1.34	85.45 ± 2.32	0.756 ± 0.186
EIEM (3)	91.66 ± 1.82	84.66 ± 3.11	0.778 ± 0.200
EIEM (2)	92.00 ± 1.57	85.22 ± 2.69	0.754 ± 0.207
EIEM (1)	91.07 ± 2.88	83.73 ± 4.75	0.838 ± 0.327

Bold values indicate the row with the best overall performance.

**FIGURE 3:**
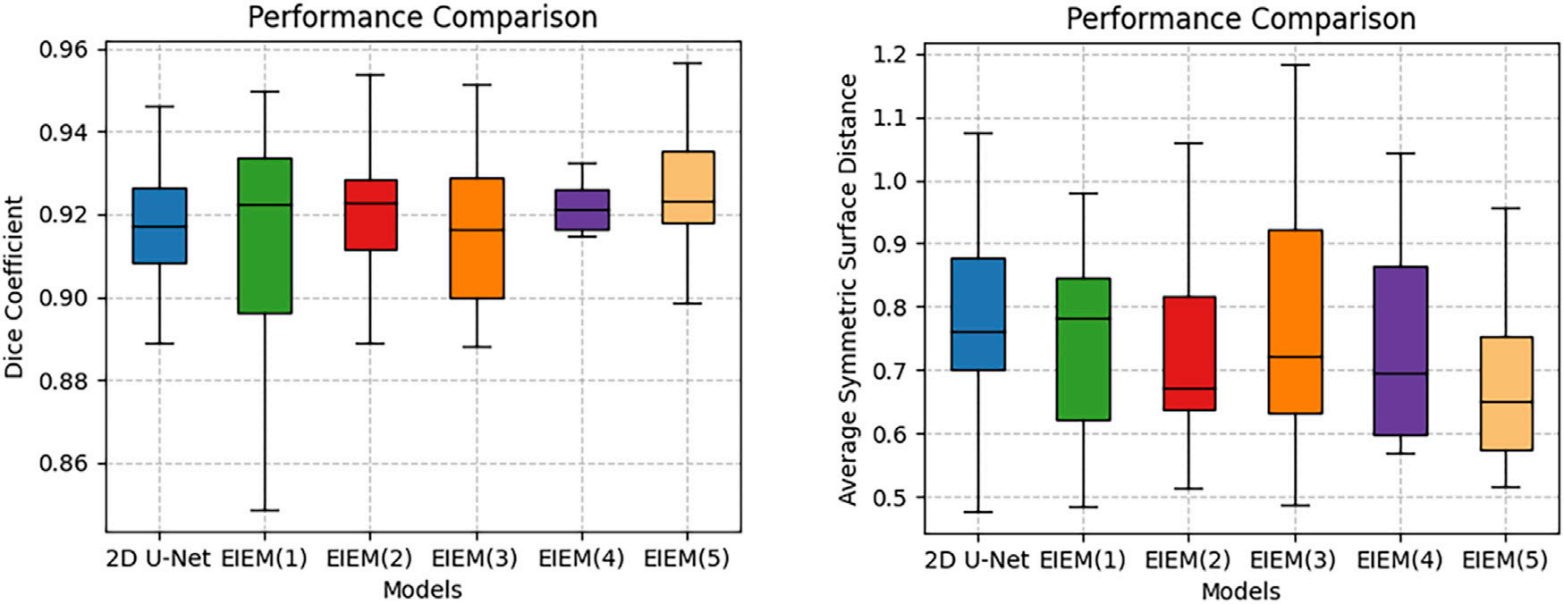
Boxplots of DC and ASSD for models with different side branch numbers.

The boxplots in Figure 3 visually represent the distribution of the Dice coefficients and average symmetric surface distances corresponding to the numerical data in Table 2. These boxplots complement Table 2 by providing a clear and comparative view of the data’s variability, making it easier to identify patterns and trends across different branch configurations, which may not be immediately apparent from the tabular data alone. In boxplots, data variability is typically illustrated by indicators such as the interquartile range (IQR) or the overall width of the distribution. A wider IQR indicates higher variability, while a narrower IQR suggests more consistent performance. Other aspects include the central tendency, which is represented by the median line in the boxplots, a robust measure that is unaffected by outliers. For instance, while integrating EIEM improves segmentation accuracy overall, particularly in capturing subtle edge details, the impact of varying branch quantities on the mean DC appears less pronounced, which could be attributed to factors like object morphology complexity, image noise, or inherent model limitations. The boxplots show how DC values are distributed for each configuration, with wider spreads indicating higher variability in segmentation performance. This variability is also evident in the higher standard deviation reported in Table 2, which reflects similar distribution patterns. However, the boxplots provide a more comprehensive view by including the IQR, which, along with the overall distribution, offers additional insights into data distribution and is more robust to outliers compared to the standard deviation. On the contrary, the number of branches significantly impacts the ASSD results, exhibiting a noticeable decrease in the mean ASSD with an increasing number of branches, reflecting better boundary accuracy. The boxplots reinforce this observation by showing a more concentrated ASSD distribution as branch numbers increase, reflecting improved consistency in boundary delineation. Although the performance improvements are not strictly linear, the overall trend shows enhancement as network complexity increases. EIEM(5) achieves the best balance between network complexity and data characteristics, delivering optimal performance across the evaluated metrics.

**FIGURE 4:**
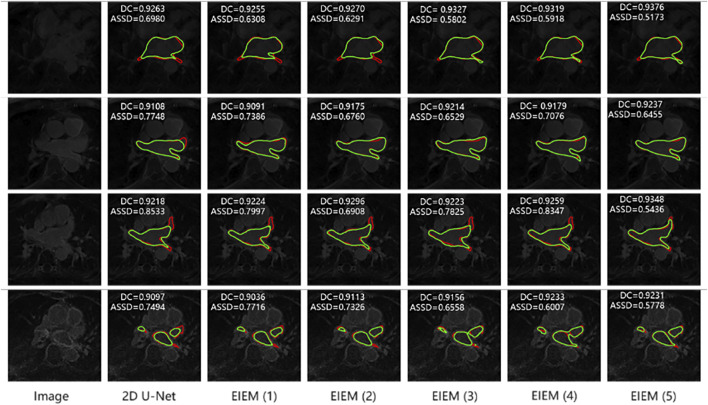
Segmentation results of models with different side branch numbers (In the top left corner are the metrics for the corresponding 3D data). Predicted edges: green; GT edges: red.

Figure 4 illustrates the visualization outcomes of models employing varying numbers of branches. For clarity, only the image edges are shown. Each row corresponds to an image along with its segmentation results from different models. GT labels are highlighted in red, while predicted images are depicted in green. EIEM branches significantly contribute to improving segmentation accuracy and enhancing edge detection. As the number of branches increases, there is a more precise delineation of object boundaries, particularly in detecting the edge of prominent terminations. This is evident from the closer alignment observed between predicted edges (green) and GT edges (red).

#### 3.2.2 The SWCE loss function

Ablation experiments were conducted to analyze the impact of different parameter settings (*a*, *b*, *c*, *d*) for the SWCE loss function on the network’s performance, as elucidated in Table 3.

**TABLE 3:** Ablation experiments investigating the optimal parameter settings for the SWCE loss function, evaluated through DC, JC, and ASSD with ± std following the values.

Parameters (*a*/*b*/*c*/*d*)	DC (%)	JC (%)	ASSD (mm)
(2/0.1/1/1)	92.26 ± 1.48	85.67 ± 2.56	0.699 ± 0.144
(4/0.1/1/1)	92.38 ± 1.42	85.87 ± 2.47	0.704 ± 0.162
(8/0.1/1/1)	92.00 ± 1.51	85.23 ± 2.58	0.764 ± 0.202
(4/0.05/1/1)	92.01 ± 1.70	85.24 ± 2.88	0.745 ± 0.195
(4/0.2/1/1)	92.12 ± 1.39	85.43 ± 2.39	0.744 ± 0.184
(4/0.1/0.5/1)	91.91 ± 1.67	85.07 ± 2.86	0.756 ± 0.188
(4/0.1/2/1)	92.23 ± 1.38	85.60 ± 2.37	0.732 ± 0.171
**(4/0.1/1/2)**	**92.43** ± **1.36**	**85.96** ± **2.35**	**0.693** ± **0.152**
(4/0.1/1/4)	92.03 ± 1.39	85.28 ± 2.40	0.704 ± 0.162

Bold values indicate the row with the best overall performance.

The parameter *a* controls the weight assigned to pixels located on the edges. To identify the optimal value for *a*, we tested a range from 2 to 8 (2, 4, 8). The results indicated that lower values (e.g., 2) did not sufficiently emphasize edge details. Conversely, higher values (e.g., 8) improved edge detection but showed diminishing returns and potential overemphasis on edge pixels. The intermediate value of 4 was found to strike the best balance. This suggests that moderate edge weighting improves the model’s ability to capture boundary details without excessive focus on the edges, thereby optimizing segmentation performance. Parameter *b* adjusts the rate at which the weight of pixels decreases with increasing distance from the edge. We evaluated values ranging from 0.05 to 0.2 (0.05, 0.1, 0.2). Smaller values (e.g., 0.05) led to a steep weight decay, focusing excessively on edge pixels and diminishing the representation of background pixels. Larger values resulted in a more gradual decay, which balanced the influence of edge and non-edge pixels but sometimes (e.g., a value of 0.2) reduced the emphasis on crucial edge features. The value of 0.1 was optimal, providing an effective balance that improved segmentation performance by adequately considering pixels both near and far from the edges. The class-wise weighting parameter *c* balances the contributions of positive and negative samples during training. Parameters *a* and *d* increase the weight of positive samples, while the weight decay mechanism reduces the weight of negative samples. Together with *c*, these factors shape how the model learns from each class. We explored values from 0.5 to 2 (0.5, 1, 2) for *c*. Lower values (e.g., 0.5) did not adequately weight positive samples, affecting the model’s ability to learn from the minority class. Higher values (e.g., 2) increased the weight of positive samples but could lead to reduced sensitivity to negative samples. The value of 1 provided the optimal balance, ensuring effective learning from both positive and negative samples and mitigating class imbalance. The parameter *d* defines the range of pixels considered around the edge, extending the annotated edge region inward. We tested values ranging from 1 to 4 (1, 2, 4) for *d*. Smaller values (e.g., 1) offered limited context, resulting in suboptimal edge feature learning. Larger values (e.g., 4) included more context but risked overexpansion, potentially introducing noise. The value of 2 was most effective, offering sufficient edge context without excessive expansion, thereby enhancing segmentation accuracy.

Based on the ablation study results, the combination of (a = 4, b = 0.1, c = 1, d = 2) achieves the best segmentation performance, with the highest DC and JC scores and the lowest ASSD. In summary, the proposed SWCE loss function effectively reflects the impact of parameters *a*, *b*, *c*, and *d* corresponding to edge weighting, weight decay rate, class-wise weighting, and edge range, enhancing the algorithm’s performance in fine-grained perception of target structures and segmentation accuracy. By selecting and tuning these parameters, the SWCE loss encourages the model to focus more on edge regions while providing flexibility for optimizing segmentation outcomes.

#### 3.2.3 Modification of edge learning components

Furthermore, additional experiments were conducted to comparatively analyze the effectiveness of the designed loss function $L_{SWCE}$ . The results of replacing the SWCE loss function with the BCE loss function are documented in the first row in Table 4. The results indicate that SWCE loss, compared to traditional BCE loss, effectively captures edge information by managing the contribution of positive and negative samples, increasing the model’s focus on boundary transition areas and reducing attention to distant regions. This enhances the precision of the algorithm in left atrial segmentation.

**TABLE 4:** Several ablation experiments on the proposed method based on DC, JC, and ASSD with ± std following the values.

Method	DC (%)	JC (%)	ASSD (mm)
EIEM (*L_BCE_*)	91.83 ± 1.78	84.95 ± 3.02	0.785 ± 0.246
EIEM (Canny)	92.09 ± 1.33	85.37 ± 2.30	0.745 ± 0.143
EIEM (w/o CA)	91.99 ± 1.49	85.21 ± 2.56	0.748 ± 0.156
EIEM (w/o oi2)	92.16 ± 1.50	85.49 ± 2.57	0.744 ± 0.166
The proposed method	**92.43** ± **1.36**	**85.96** ± **2.35**	**0.693** ± **0.152**

Bold values indicate the row with the best overall performance.

Next, as shown in Table 4, experiments were conducted to investigate the impact of different methods on the overall segmentation performance, such as extracting GT edges (the second row), utilizing channel attention in the edge-learning branches (the third row), and deleting edge information in the skip connections (the fourth row). The results indicate that the erosion-based method employed in this study outperforms the Canny edge detection method for edge extraction. This superiority could be attributed to the erosion-based method’s ability to more accurately capture valid edges, thus enhancing segmentation accuracy. Additionally, in the absence of channel attention, there is a degradation in DC, JC, and ASSD metrics compared to the proposed method since channel attention helps the model focus better on crucial features, thereby improving segmentation precision. Furthermore, when all oi2 outputs are removed from the EIEM module, there is a deterioration in performance. This suggests that the lack of edge information in skip connections may impair segmentation performance.

**FIGURE 5:**
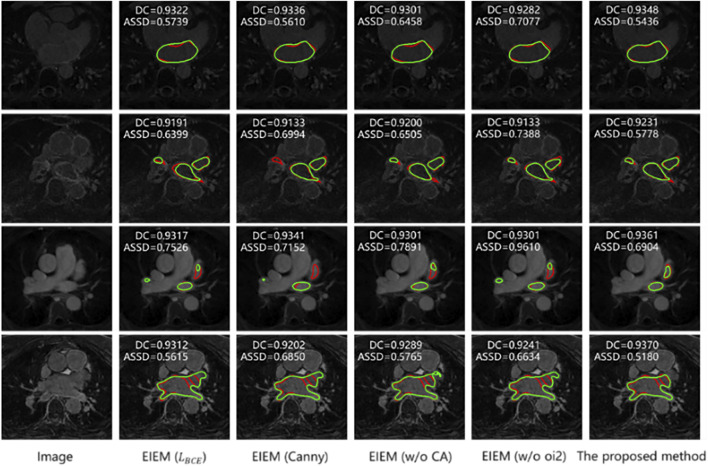
Segmentation results of models with various component replacements (In the top left corner are the metrics for the corresponding 3D data). Predicted edges: green; GT edges: red.

In Figure 5, each row presents a sample from the testing dataset along with its corresponding GT edge (highlighted in red) and segmentation results from various models, with predicted edges highlighted in green. Consistent with the results in Table 4, the samples in Figure 5 illustrate that the method proposed in this study outperforms counterparts utilizing the BCE loss, employing the Canny-based method for edge extraction, lacking channel attention, and not incorporating edge information in the skip connections. Notably, when there is only one detection target with a relatively regular shape, the improvement in segmentation performance by the proposed method is minimal (as shown in the first row). However, when multiple detection targets are present in an image, the detection capability of this method is notably enhanced (as depicted in the second, third, and fourth rows). Nevertheless, when detection targets are irregular and multiple detection regions exist in an image, although the proposed method shows some improvement, it cannot accurately identify the segmented targets and their edges.

### 3.3 Generalization capability

To evaluate the generalization performance of the proposed framework, we validated it on the MSD heart dataset. When the model was tested directly on the new dataset, the DC was 0.8, suggesting a reasonable level of adaptability under challenging conditions. This initial result prompted further experimentation to enhance the segmentation performance. The dataset is characterized by a limited number of samples and large variability. Given these factors, we simplified the model by reducing the EIEM structure from five to four branches while keeping all other parameters unchanged. This modification aimed to ensure better convergence and improve the model’s stability on new data by reducing complexity, as combining a small dataset with a relatively complex model can hinder effective optimization and lead to non-convergence. Table 5 compares the results of the proposed model with the U-Net backbone on the MSD heart dataset. The proposed model consistently outperformed the U-Net backbone across all evaluation metrics: DC, JC, and ASSD. The DC, a key measure of segmentation accuracy, improved from 0.88 with the U-Net backbone to 0.92, demonstrating that the proposed model was more effective at correctly identifying and segmenting the LA. Similarly, the JC showed a 6% improvement, suggesting better performance in terms of overlap between predicted and GT regions. Moreover, the ASSD was reduced by 43%, from 2.27 mm to 1.28 mm, indicating a significant enhancement in boundary precision, with the proposed model showing better alignment between predicted and actual LA boundaries. The reduced ASSD is particularly important in medical image segmentation, where fine-grained accuracy at the boundaries can have a meaningful impact on clinical decisions. Even on the smaller MSD heart dataset, the model surpassed the U-Net backbone, showing improvements in both segmentation accuracy and boundary precision. These findings underscore the robustness and adaptability of the proposed framework, highlighting its potential for effective application across diverse clinical datasets.

**TABLE 5:** Performance comparison between the proposed model and U-Net backbone on the MSD heart dataset based on DC, JC, and ASSD with ± std following the values.

Method	DC	JC	ASSD (mm)
U-Net Backbone	0.88 ± 0.03	0.79 ± 0.04	2.27 ± 0.97
Proposed EIEM (4)	0.92 ± 0.01	0.85 ± 0.01	1.28 ± 0.14

## 4 Discussion

### 4.1 Capacity for edge information learning

In previous segmentation algorithms, the absence of constraints on edge information often resulted in inaccurate and unstable segmentation outcomes. This is because edge information carries crucial geometric and semantic indications in images, and the lack of constraints on edges can lead to imprecise boundary delineations. Hence, incorporating edge constraints is essential for improving the accuracy of atrial segmentation. The proposed EIEM, designed as a tributary structure of the network, explicitly processes edge information independently of other image features. On one hand, the edge features extracted by the edge information blocks are directly applied to the learning of target edges, constraining the network’s segmentation outputs with edge information. On the other hand, in combination with skip connections in the target segmentation task, the backbone network considers edge information constraints while learning region features, leading to better delineation of regions and, consequently, enhanced segmentation precision. Moreover, multiple side branches for edge feature learning extend the module’s capacity to capture multi-scale features, thereby further improving segmentation accuracy. The reason behind this lies in the complementary nature of multi-scale features in capturing edge information across different levels of detail. By fusing features from multiple scales, the network gains a more comprehensive understanding of edge characteristics, allowing it to better constrain segmentation outputs with accurate edge information. Consequently, the inclusion of multi-scale feature fusion not only enriches the representation of edge information but also strengthens its capacity to guide the segmentation process effectively, ultimately leading to superior segmentation precision.

The incorporation of edge information enhancement leads to a notable improvement in the ASSD metric. By the explicit utilization of edge features for guiding the segmentation process, our proposed method ensures that the network is better equipped to accurately delineate target boundaries within the MRI images. Consequently, the segmentation outputs exhibit reduced surface distance discrepancies between the predicted and GT boundaries, resulting in a lower ASSD metric. On the other hand, the DC primarily measures the overlap between the segmented regions and the GT without explicitly considering boundary delineation. Therefore, algorithms that solely optimize for region overlap may achieve similar Dice scores despite potential differences in boundary accuracy. However, our method’s utilization of edge information ensures more precise boundary localization, which may not significantly impact the DC but ensures a more accurate delineation of boundaries.

### 4.2 Enhanced edge constraints: the SWCE loss function

To further enhance the learning of edge information, this study proposed a novel SWCE loss function. The SWCE loss function imposes constraints on the network’s learning of edge information through several aspects: edge weighting, weight decay mechanism, and edge range. Primarily, the edge weighting directs the model’s attention towards the boundaries of the target region, thereby augmenting its sensitivity to contours and shapes. Subsequently, the weight decay mechanism allows adaptable modifications to pixel weights based on the distance between pixels and edges during loss calculation. Pixels farther from the edges are assigned lower calculation weights compared to those closer to the edges, thereby reducing excessive focus on regions distant from the target. Finally, adjusting the edge range means extending the annotated edge region inwards, augmenting from one layer of target pixels to multiple layers, thus enlarging the edge detection target and providing richer information for supervision.

The erosion-based approach is a reliable technique utilized for extracting GT edges. This method functions by shrinking the target area within an image layer by layer, obtaining the target edge with the specified number of layers inside the original target region, thereby achieving accurate edge delineation. Channel attention modules dynamically adjust feature responses across different channels, enabling the edge-learning branches to focus on edge-related information while suppressing irrelevant details. This selective enhancement of critical features contributes to improving the network’s ability to discern edge variations and accurately delineate target boundaries. Furthermore, incorporating edge information into the skip connections of the network enhances a contextual understanding of the target edges, facilitating better feature propagation and integration across different network layers and enabling more effective utilization of edge-related information throughout the network.

In summary, the SWCE loss function and these edge learning components contribute to the network’s capability to accurately delineate target boundaries and produce high-quality segmentation results.

### 4.3 Class imbalance in medical images

In medical image segmentation, class imbalance is a common challenge, where certain classes appear more frequently in images than others. This imbalance can cause deep learning networks to bias towards majority classes during the learning process, overlooking important information from minority classes and ultimately affecting the accuracy of segmentation results. In the segmentation of LA in MRI images, the relatively small LA region in many slices results in suboptimal segmentation outcomes, particularly along boundaries where precision is crucial.

To address this challenge, the pre-processing step initially involves image cropping to increase the proportion of the target region and reduce the impact of class imbalance. However, imbalance may persist even after cropping, especially for edge segmentation tasks. To overcome this, our study introduces the SWCE loss function, which dynamically balances the contribution of positive and negative samples in pixel-wise loss calculation by assigning weights. Parameter *d* (edge range) expands the edge region by extending layers inward from the annotated boundary, increasing the number of pixels labeled as the target during training. Meanwhile, parameter *a* (edge weighting) directly increases the weight of these edge pixels in the loss calculation. Together, these adjustments provide the model with more edge information and emphasize boundary learning, improving its ability to capture edge features that might otherwise be under-represented due to class imbalance. Additionally, the weight decay mechanism controls the reduction of pixel weights based on their distance from the edge. Negative samples are assigned lower weights in the loss calculation, effectively reducing their contribution and allowing the model to focus on more critical boundary regions. On the foundation of these spatial adjustments, parameter *c* (class-wise weighting) further balances the contributions of positive and negative samples globally, ensuring that the network does not become biased toward the majority class (background) and can effectively learn from both positive and negative samples. This helps prevent the model from being dominated by negative samples while maintaining attention to the target structures.

Through this combination of mechanisms, the SWCE loss function effectively mitigates class imbalance and enhances the network’s capability to handle imbalanced data. This leads to more accurate and reliable segmentation results.

### 4.4 Clinical implications and applications

Precise segmentation plays a critical role in cardiac imaging. The enhanced segmentation accuracy achieved by our method holds substantial clinical significance, particularly for cardiac conditions involving the left atrium, such as atrial fibrillation. Accurate delineation of atrial boundaries is essential in procedures like radiofrequency ablation, where it guides catheter placement and ablation path planning, ultimately contributing to safer and more effective interventions. Additionally, accurate boundary delineation not only improves the reliability of automated measurements but also minimizes the need for manual adjustments, thereby optimizing diagnostic processes and increasing efficiency. The incorporation of this advanced segmentation technique into clinical practice has the potential to enhance various aspects of patient care, from more accurate disease monitoring to better assessment of treatment response. These improvements can ultimately contribute to better patient outcomes and more efficient healthcare delivery.

### 4.5 Limitations and future work

Although this study offers valuable insights into LA segmentation, several limitations should be acknowledged.

One limitation of the study is its utilization of a 2D architecture for the segmentation task. Although this approach presents advantages in computational efficiency and simplicity, it might fail to capture certain spatial and structural complexities inherent in three-dimensional data. Specifically, the 2D framework processes each slice independently, which can result in the loss of continuity and contextual information between adjacent slices, limiting segmentation accuracy. Future research could investigate incorporating 3D architectures, such as 3D U-Net, which are better suited for handling volumetric data. By leveraging 3D models, continuous edge information can be extracted across multiple slices in three-dimensional space, allowing for more effective delineation of left atrial structural details during segmentation. This would potentially enhance the accuracy and robustness of the segmentation process, facilitating more precise clinical applications.

Furthermore, incorporating additional modules like the EIEM and the customized loss function increases the computational complexity of the segmentation framework. In the experiments, we observed that the simplest model (backbone) contains approximately 10M trainable parameters, while the most complex configuration contains around 23M. This increase in network complexity led to longer training time per epoch, with the most complex configuration requiring approximately 2.3 times the training time compared to the backbone model. The additional trainable parameters result in greater computational demands and potentially longer inference times, which could impact both training and deployment phases. Thus, striking a balance between computational efficiency and segmentation performance becomes crucial when considering the model’s applicability in clinical practice. Further optimization efforts should focus on reducing computational overhead, such as through model compression or efficient inference strategies, while maintaining segmentation quality.

Moreover, while our method has shown promise in left atrial segmentation, it is important to acknowledge that the improvement in DC may not be as significant as desired. One notable factor contributing to this limitation is the challenge of dealing with irregular shapes and discrete multiple targets, which are common in left atrial imaging. In such cases, our algorithm encounters difficulties in accurately delineating edge information, resulting in suboptimal segmentation outcomes. This challenge may arise from the algorithm’s dependence on specific features or characteristics of the data, which insufficiently model the complex and variable anatomical structures, especially in cases where the left atrial anatomy exhibits high variability. To address this limitation, future research could explore integrating more advanced feature extraction techniques or employing machine learning algorithms capable of capturing subtle variations and irregularities in shape, such as transformer-based architectures or deformable convolution networks.

Therefore, while our method demonstrates advancements in left atrial segmentation, the identified shortcomings underscore areas for further refinement. Addressing these challenges through methods such as the integration of 3D architectures, optimization of computational complexity, and improvements in handling irregular structures will be valuable for future research in atrial segmentation and broader applications in medical image analysis.

## 5 Conclusion

In summary, this study presents a novel approach to image segmentation geared towards the challenges associated with class imbalance and the segmentation of blurred edges in medical images. On one hand, the introduced EIEM not only extracts edge features to directly guide edge segmentation but also indirectly constrains the network’s learning of target shapes. On the other hand, the proposed SWCE loss function not only constrains the edge learning from aspects such as edge weighting, weight decay mechanism, and edge range but also addresses the common class imbalance issue in medical images through adjusting the weights of positive and negative samples in loss calculation. The integration of these two mechanisms significantly improves the segmentation accuracy of left atrial MRI images. The findings indicate the substantial potential of our method for clinical applications, such as improving diagnostic accuracy and treatment planning, particularly in addressing the challenge of blurred and irregular edges, which may lead to more reliable assessments and better patient outcomes. Future work will involve testing and optimizing the method on a broader range of datasets and clinical scenarios to further validate its robustness and effectiveness. Additionally, its applicability to other anatomical structures and imaging modalities will be explored to establish a more comprehensive and clinically impactful segmentation strategy.

## Data Availability

Publicly available datasets were analyzed in this study. This data can be found here: https://www.cardiacatlas.org/atriaseg2018-challenge/atria-seg-data https://drive.google.com/drive/folders/1HqEgzS8BV2c7xYNrZdEAnrHk7osJJ--2.
